# Gender and depression: dual pathways influencing adolescents' physical activity and psychological wellbeing

**DOI:** 10.3389/fpubh.2025.1663388

**Published:** 2025-10-17

**Authors:** Dan Bai, Mingdong Wu, Ying Pang, Xiaoming Liu

**Affiliations:** ^1^School of Physical Education, Hunan Normal University, Changsha, China; ^2^School of Physical Education, Guizhou Education University, Guizhou, China; ^3^Faculty of Educational Sciences and Technology, Universiti Teknologi Malaysia, Johor Bahru, Johor, Malaysia

**Keywords:** gender differences, depression, physical activity, psychological wellbeing, adolescents

## Abstract

**Objectives:**

This study investigates the influence of gender and depression on physical activity (PA) and psychological wellbeing among adolescents in Shanghai.

**Methods:**

A purposive offline survey conducted from June to December 2023 collected data from 416 participants (216 boys, 200 girls). This study employed SPSS software version 29 to conduct descriptive statistics and multivariate analysis of variance (MANOVA).

**Results:**

The findings revealed a significant gender difference in moderate-to-vigorous physical activity (MVPA), with boys reporting higher levels than girls (η^2^ = 0.05). However, gender did not significantly affect psychological variables, including academic self-efficacy (ASE), mindfulness (MD), emotional intelligence (EI), self-esteem (SE), and social support (SS). Depression levels had a significant impact on all psychological variables, with students experiencing low depression scoring higher on ASE, MD, EI, SE, and SS compared to those with high depression (η^2^ = 0.11–0.25). Conversely, depression levels did not significantly influence MVPA.

**Conclusions:**

These results emphasized the critical role of addressing depression to improve adolescents' psychological wellbeing while highlighting the need for gender-specific interventions to encourage physical activity (PA).

## 1 Introduction

Adolescence is a critical developmental period characterized by rapid physical, emotional, and psychological changes. During this stage, factors such as gender, physical activity (PA), and mental health can play a crucial role in shaping PA and psychological outcomes (1). Depression, in particular, is a prevalent mental health concern among adolescents, impacting their academic performance, social interactions, and overall wellbeing (1). Understanding the intricate relationships between these factors is essential for developing effective interventions to promote adolescent health and wellbeing.

Gender differences in PA levels have been a prominent focus in recent research. Boys tend to engage in higher levels of moderate-to-vigorous physical activity (MVPA) compared to girls, with greater variability observed in boys' activity levels. These findings underscore the importance of examining the distribution of PA in future studies to better understand the underlying dynamics driving these disparities (2). Additionally, perceived stress has been shown to negatively impact the frequency of PA among adolescents, regardless of gender. Stratified multivariate analyses reveal that higher levels of perceived stress are consistently associated with lower PA participation over the past 6 months in both males and females (3). Gender differences in PA are particularly evident during the junior high school years, as Telford et al. (4) highlighted, their research indicates that gender plays a significant role in shaping PA behaviors, with boys generally reporting higher activity levels than girls. This disparity highlights the need for targeted interventions to address the lower levels of PA observed among adolescent girls.

Studies also have highlighted both similarities and differences between males and females in genetic, phenotypic, personal, social, cultural, and emotional domains. In terms of emotional abilities, females have been shown to excel in recognizing others' emotions, demonstrating greater perceptiveness and empathy, and experiencing both positive and negative emotions more intensely than males (5, 6). Specifically, adolescent girls outperform boys in ability-based emotional intelligence (EI), whereas boys score higher in emotional self-concept across age groups. Furthermore, boys tend to overestimate their emotional abilities, while girls, particularly adolescents, tend to underestimate them. Girls also consistently score higher in meta-emotional beliefs compared to boys (7).

The role of social support (SS) in gender differences has also been extensively studied. Online SS has been found to negatively correlate with depressive symptoms, with SE serving as a mediator. Gender moderates the relationship between online SS and self-esteem (SE), with the effect being more pronounced among males (8). Moreover, females are more likely to seek and benefit from SS, as its positive effects on SE and life satisfaction are stronger for females than for males (9). However, the relationship between gender and mental health varies significantly across regions, countries, and cultural contexts. In high-gender-equality countries, gender disparities in mental health may reflect complex and sometimes incongruent patterns between societal expectations and individual realities (10).

Gender differences in stress coping strategies have also drawn attention. Research indicates that males exhibit higher levels of internal locus of control and resilience, whereas females demonstrate lower SE (11). While no significant differences have been observed in overall coping styles, females tend to favor emotion-focused strategies, such as seeking SS or engaging in distraction activities (12). Additionally, the interaction between the quality of SS and gender significantly predicts perceived stress, with notable variations in how males and females manifest stress in their relationships (13). Research also has shown that girls have much lower mathematics self-efficacy than boys, which could contribute to the underrepresentation of women in science, technology, engineering, and mathematics STEM fields (14). A systematic review also emphasizes the importance of considering gender differences in ASE to promote female representation in education (15).

The existing literature highlights the role of gender in moderating the protective effects of mindfulness (MD). For instance, research has shown that the protective effect of MD on depression is more pronounced in females than in males (16). Furthermore, prior studies have reported that males tend to score higher than females on specific MD facets, such as Awareness and Nonjudge (17). Recent findings by Cao et al. (18) emphasized the moderating effects of gender on MD facets, underscoring the need for gender-specific MD interventions aimed at improving sleep quality and managing stress. In summary, this body of research underscores the significance of focusing on present physical sensations as a core MD practice. This approach helps individuals detach from arousal triggered by daily stress, thereby alleviating stress-induced sleep disturbances. Consequently, these findings advocate for the development of tailored interventions and gender-specific strategies to maximize the efficacy of MD practices (18).

Depression encompasses a cluster of negative emotional states and behavioral manifestations, including feelings of worthlessness and loneliness, frequent crying, excessive worry about misbehavior, and a compulsive need for perfection (19). It is widely regarded as a hallmark of poor psychological adjustment, posing significant threats to individuals' interpersonal relationships, social functioning, and overall quality of life, while also elevating the risk of suicide (20). A growing body of empirical evidence suggests that PA is inversely associated with depressive symptoms among adolescents (21–24). But recent studies have suggested that rather than PA leading to depression, it may be the case that depression contributes to reduced PA levels (25). Sowislo and Orth (25) conducted a longitudinal study spanning 11 years, involving four waves of assessment, to investigate changes in PA, screen time (ST), depressive symptoms, and anxiety symptoms during adolescence. The study further explored the bidirectional relationships between initial levels of PA, ST, and symptoms of depression and anxiety as predictors of subsequent changes in one another. Findings revealed that increases in anxiety were associated with elevated ST and heightened depressive symptoms. Importantly, higher baseline levels of depressive symptoms significantly predicted subsequent reductions in PA. These results challenge the traditional unidirectional perspective and suggest that mood disorders may impair motivation and energy, thereby hindering adolescents' engagement in physical activity (25).

Depression, as a significant psychological factor, exerts considerable influence on various mental health outcomes (26). Defined by persistent feelings of sadness or emptiness lasting most of the day for at least 2 weeks, depression often fosters a sense of hopelessness and worthlessness, which profoundly impacts an individual's emotional state and overall outlook on life (26). One hallmark symptom of depression is anhedonia—the inability to derive pleasure from activities that were previously enjoyable. This loss of interest often leads to diminished participation in social interactions and recreational activities, further reinforcing a sense of isolation and detachment (27). Depression also negatively affects cognitive processes, impairing focus, decision-making, and memory. These cognitive disruptions can significantly impact academic and occupational performance, further compounding the challenges faced by individuals with depression (26).

Individuals with depression are prone to engaging in negative self-perceptions and cognitive distortions, often adopting a pessimistic view of their abilities and future. Such cognitive biases contribute to feelings of inadequacy, self-doubt, and despair, perpetuating the depressive state (27). Motivational deficits are another defining characteristic of depression, leading to decreased engagement in everyday activities, including work, education, and social events. This inactivity often initiates a self-reinforcing cycle, where reduced participation worsens depressive symptoms, further lowering motivation (28). Depression frequently results in social withdrawal, with individuals distancing themselves from family and friends. The reduction in social interactions and the erosion of support networks can amplify feelings of loneliness, further exacerbating the condition (26). Depression is also strongly associated with low SE. Affected individuals often struggle with feelings of unworthiness or disproportionate guilt over perceived shortcomings, leading to a diminished sense of self-worth and a persistently negative self-image (25).

During adolescence, PA not only plays a critical role in physical health but also significantly impacts psychological wellbeing. However, gender differences and depression levels may modulate both the participation in and the effects of PA. Additionally, psychological factors such as ASE, MD, EI, SE, and SS are vital for enhancing adolescents' psychological resilience, stress management, and personal growth. By examining the relationships among these variables, the study aims to uncover how depression negatively impacts adolescents' psychological wellbeing and how strengthening these psychological factors can mitigate depressive symptoms. Moreover, understanding the role of gender differences in PA and psychological factors provides a foundation for designing personalized interventions that effectively address the diverse needs of adolescents. Thus, this research not only seeks to fill gaps in existing literature but also aims to provide scientific evidence for developing multidimensional, targeted health promotion interventions, enabling adolescents to achieve optimal development in both mental health and PA.

Therefore, the primary objective of this study is to examine the influence of gender and depression on PA and psychological wellbeing among adolescents in Shanghai, with a particular focus on how these factors are associated with ASE, MD, EI, SE, and SS.

## 2 Materials and methods

### 2.1 Participants

This study employed a cross-sectional survey design to investigate the influence of gender and depression on PA and psychological wellbeing among adolescents in Shanghai. This study was conducted among junior high school students in Shanghai from semester, 2023 to January, 2024 during the academic year. Participants were recruited through purposive sampling. Three junior high schools were selected from both urban and suburban districts of Shanghai, including Jing'an, Hongkou, and Xuhui (urban) and Minhang, Qingpu, and Jinshan (suburban), with one school chosen from each district. The participating schools were intentionally selected because they possess typical characteristics of junior high schools in Shanghai in terms of educational management, curriculum structure, student population size, and demographic composition. Within each selected school, one sixth-grade and one seventh-grade class were randomly sampled to participate in assessments of PA, EI, SS, MD, ASE, SE, and depression. This study was approved by the Ethics Committee of Hunan Normal University. Before distributing the questionnaire, the students and their parents agreed and signed the informed consent form. To prevent confusion between physical activity and school-based physical education, all students in the selected classes were asked to complete the questionnaire about PA, ASE, MD, EI, SE, SS and their age. A total of 432 responses were collected, of which 416 were deemed valid. Among the valid responses, 216 were male and 200 were female. The average ages of male, female, and total participants were 12.41, 13.29, and 13.35 years, respectively.

### 2.2 PA

In this study, PA referred to all physical and sports activities that junior high school students might be involved in as one of the healthy lifestyles to adopt, whether on or off campus. Participants' PA was assessed by frequency in a typical week (ranging from 0 to 8) in which they engaged in light physical activity (LPA), moderate physical activity (MPA), and vigorous physical activity (VPA) during leisure time for at least 15 min. Based on the scoring guidelines (29), the three intensity levels of PA were based on the corresponding metabolic equivalents of Task (MET) values (LPA = 3 MET, MPA = 5 MET, and VPA = 9 MET). Precisely, the PA intensity level was calculated as follows: LPA = frequency × 3 metabolic equivalents (MET), MPA = frequency × 5 MET, and VPA = frequency × 9 MET. The calculated scores for MVPA = MPA (range, 0–40) + VPA (range, 0–72) were used for data analysis. We translated the questionnaire into Chinese, before distributing the questionnaires, the questionnaires passed the content validity and translate validity by two experts in China.

### 2.3 Mindfulness

In this study, MD is a mental state in which students focus their awareness on daily life. The Mindfulness Attention Awareness (MAAS) (30) was applied to assess the MD symptoms among participants. In view of the situation and particularity of junior high school students, this research modified some items and deleted some items with low outer loading through a pilot study. Participants responded to items regarding their MD state in daily life, such as “I find it difficult to consistently focus on what I'm doing on what happened, I wasn't quite aware of what I was doing…” The 4-point Likert scale used for each statement ranged from 0 (not at all) to 3 (all of the time), and the average score of the six items was calculated for data analysis. Before distributing the questionnaires, the questionnaires passed the content validity and translate validity by two experts in China, all items' factor loading was above 0.6, CR was 0.799, AVE was 0.486, and CA was 0.786.

### 2.4 Academic self-efficacy

The 4-item questionnaires were adapted to assess participants' ASE academic (31), and some amendments were made regarding the class situation of junior high school students in Shanghai. Participants responded to items regarding how often they get good performance/fail in class and school; they were asked to rate five items using a 4-point Likert-type scale ranging from 0 (none of the time) to 3 (all of the time). Such as “I am confident in my ability to learn, I can focus on studying subjects, I can master the knowledge points taught by the teacher…” The average score of these five items was calculated for data analysis. Before distributing the questionnaires, the questionnaires passed the content validity and translate validity by two experts in China, all items' factor loading was above 0.6, CR was 0.906, AVE was 0.659, and CA was 0.87, that can be indicated the questionnaires had relatively good reliability and validity.

### 2.5 Social support

In this study, SS referred to junior high school students obtaining support from family, friends, and significant others. The Multidimensional Scale of Perceived Social Support (MSPSS) (32) was applied to assess the SS symptoms among participants, the Cronbach's alpha of original scale was 0.88. In view of the situation and particularity of junior high school students in Shanghai, we modified some items and translated into Chinese. Participants responded to items regarding how often they obtain support from family, friends, and significant others. We deleted some items of low outer loading through pilot study or items with similar meanings. The last scale in this research contained six items, they were: “My family really tries to help me, I get the emotional help and support I need from my family, My friends really try to help me, I can count on my friends when things go wrong, I can talk about my problems with my family, I have friends with whom I can share my joys and sorrows.” The four-point Likert scale that was used for each statement ranged from 0 (not at all) to 3 (all of the time). Before distributing the questionnaires, the questionnaires passed the content validity and translate validity by two experts in China, all items' factor loading was above 0.6, composite reliability (CR) was 0.845, the average extracted (AVE) was 0.545, and the Cronbach's alpha (CA) was 0.834, that can be indicated the questionnaires had relatively good reliability and validity.

### 2.6 Emotional intelligence

In this study, EI including self-emotion appraisals, others' emotion appraisals, regulation of emotion and use of emotion. The Wong and Law Emotional Intelligence Scale (33) was applied to assess the EI symptoms among participants, the CA values of original scale typically ranging between 0.80 and 0.90. In view of the situation and particularity of junior high school students in Shanghai, we modified some items and translated into Chinese. Participants responded to items regarding the state of perceiving, understanding, managing, and utilize emotions on daily life. We deleted some items of low outer loading through pilot study or items with similar meanings. The last scale in this research contained eight items, they were: “I have a good sense of why I have certain feelings most of the time, I have a good understanding of my own emotions, I really understand what I feel, I am a good observer of others' emotions, I always tell myself I am a competent person, I would always encourage myself to try my best, I am able to control my temper and handle difficulties rationally, I am quite capable of controlling my own emotions.” The four-point Likert scale that was used for each statement ranged from 0 (not at all) to 3 (all of the time). Before distributing the questionnaires, the questionnaires passed the content validity and translate validity by two experts in China, all items' factor loading was above 0.6, CR was 0.884, AVE was 0.513, and CA was 0.862, that can be indicated the questionnaires had relatively good reliability and validity.

### 2.7 Self-esteem

In this study, SE was typically understood as an individual's overall subjective evaluation of their own worth or value. The Rosenberg Self-Esteem Scale (34) was applied to assess the SE symptoms among participants, the CA values of original scale typically ranging between 0.77 and 0.88. In view of the situation and particularity of junior high school students in Shanghai, we modified some items and translated into Chinese. Participants responded to items regarding how often they understood overall subjective evaluation of their own worth or value. We deleted some items of low outer loading through pilot study or items with similar meanings. The last scale in this research contained six items, they were: “I feel like I am a valuable person, at least equal to others. I think I have many advantages. All in all, I tend to consider myself a loser. I have a positive attitude toward myself. Overall, I'm happy with myself. I often feel useless.” The four-point Likert scale that was used for each statement ranged from 0 (not at all) to 3 (all of the time). Before distributing the questionnaires, the questionnaires passed the content validity and translate validity by two experts in China, all items' factor loading was above 0.6, CR was 0.881, AVE was 0.554, and the CA was 0.834, that can be indicated the questionnaires had relatively good reliability and validity.

### 2.8 Depression

The depression anxiety stress scales (DASS)-depression subscale (35) was applied to assess the depressive symptoms among participants, the CA was 0.91. In view of the situation and particularity of junior high school students in Shanghai, we modified some items and translated into Chinese. Participants responded to items regarding how often they have felt or behaved during the past week. The last scale in this research contained seven items, they were: “I feel like life is meaningless, I feel like I have nothing to look forward to, I can't seem to experience any positive feelings, I can't be enthusiastic about anything, I feel like I have no value, I feel frustrated and depressed, I find it difficult to initiate things.” The four-point Likert scale that was used for each statement ranged from 0 (not at all) to 3 (all of the time). Before distributing the questionnaires, the questionnaires passed the content validity and translate validity by two experts in China, all items' factor loading was above 0.6, CR was 0.896, AVE was 0.611, and CA was 0.891, that can be indicated the questionnaires had relatively good reliability and validity.

### 2.9 Data analysis technique

This study employed SPSS software version 29 to conduct descriptive statistics and multivariate analysis of variance (MANOVA). The primary objective of the study was to investigate whether gender and depression exhibit significant differences in PA, EI, SS, MD, ASE and SE among junior high school students in Shanghai.

## 3 Results

Table 1 presents the demographic characteristics of the respondents, including gender and age distribution. Descriptive statistics, specifically frequencies and percentages, were employed to analyze the participants' profiles. As indicated in Table 1, the sample comprised 216 male students (51.9%) and 200 female students (48.1%). Regarding age distribution, 57 students (13.7%) were 12 years old, 169 students (40.6%) were 13 years old, 177 students (42.5%) were 14 years old, and 13 students (3.2%) were 15 years old.

**Table 1:** Demographic characteristics of the respondents among Shanghai junior high school.

**Demographic**	**Category**	**Frequency**	**Percentage (%)**
Gender	Male	216	51.9
	Female	200	48.1
Age	12	57	13.7
	13	169	40.6
	14	177	42.5
	15	13	3.1

Among our various data distributions, skewness and kurtosis ranged from −0.807 to 0.379 and −0.616 to 0.843, suggesting that all variables were approximately normally distributed. The MANOVA model for psychological variables revealed a significant main effect of depression (Wilks' Lambda = 0.326, *F* = 104.54, *p* < 0.001, η^2^ = 0.674). Compared to high depression, low depression reported significantly higher levels of ASE (M = 1.94 vs. M = 1.39, η^2^ = 0.13), MD (M = 2.37 vs. M = 1.91, η^2^ = 0.15), EI (M = 2.00 vs. M = 1.52, η^2^ =0.12), SE (M = 2.07 vs. M = 1.32, η^2^ = 0.25), SS (M = 2.15 vs. M = 1.66, η^2^ = 0.05). The effect sizes η^2^ (partial eta squared) ranged from 0.11 to 0.25, indicating medium to large effects of depression levels on psychological variables (36). But the depression levels showed no significant difference in MVPA, *F*_(1, 414)_ = 1.75, *p* > 0.05, η^2^ = 0.00. The MANOVA model for gender revealed that there was a significant gender difference in MVPA, with boys (M = 57.51, SD = 26.67) reporting higher levels of PA than girls (M = 45.35, SD = 21.50), *F*_(1, 414)_ = 20.89, *p* < 0.01, η^2^ = 0.05. This indicated a small but significant effect of gender on PA. For ASE, MD, EM, SE, and SS, no significant differences were found between boys and girls (*p* > 0.05). This suggested that gender did not play a significant role in these psychological variables. The MANOVA model for interaction effects (gender^*^depression level) showed no significant interaction effects were found between gender and depression levels for any of the variables, including MVPA and psychological outcomes (*p* > 0.05). This suggested that the impact of depression on psychological and PA is consistent across genders (Table 2).

**Table 2:** MANOVA results of PA and psychological by gender and depression (*N* = 416).

**Variables**	**Total (*n* = 416) M (SD)**	**Boys (*n* = 216) M (SD)**	**Girls (*n* = 200) M (SD)**	***F***	**η2 *P***	**Low DP (*n* = 303) M (SD)**	**High DP (*n* = 113) M(SD)**	***F***	**η2 *P***	**Gender** ^*^ **DP level**	
										***F***	η**2 P**
MVPA	51.66 (25.04)	57.51 (26.67)	45.35 (21.50)	20.89^**^	0.05	52.82 (24.31)	48.58 (26.78)	1.75	0.00	0.05	0.00
ASE	1.79 (0.68)	1.83 (0.67)	1.75 (0.68)	0.55	0.00	1.94 (0.65)	1.39 (0.57)	61.63^**^	0.13	0.01	0.00
MD	2.25 (0.53)	2.25 (0.50)	2.24 (0.55)	0.07	0.00	2.37 (0.45)	1.91 (0.58)	73.09^**^	0.15	0.65	0.00
EI	1.87 (0.63)	1.88 (0.64)	1.85(0.62)	0.02	0.00	2.00 (0.60)	1.52 (0.56)	54.94^**^	0.12	0.00	0.00
SE	1.86 (0.66)	1.92 (0.64)	1.81 (0.69)	1.62	0.00	2.07 (0.57)	1.32 (0.58)	139.22^**^	0.25	0.04	0.00
SS	2.02 (0.67)	2.00 (0.68)	2.04 (0.66)	0.04	0.00	2.15 (0.63)	1.66 (0.63)	51.34^**^	0.11	1.99	0.01

^**^p < 0.01, SS, social support; SE, self-esteem; EI, emotional intelligence; MD, mindfulness; PA, physical activity; ASE, academic self-efficacy; DP, depression; M, mean; SD, standard deviation.

## 4 Discussion

In this research, a significant gender difference was found for PA, with boys engaging in more MVPA than girls. This effect, while statistically significant, was small (η^2^ = 0.05). The findings of this study were in lined with the previous research revealed that PA levels of junior high school students can be influenced by gender (4). Research has shown that there are gender differences in PA levels among youth, with girls being less PA than boys (4). Factors such as participation in organized sports, SS, and enjoyment in physical education can contribute to this gender disparity (4). Walker et al. (37) conducted research in 67 schools and 15,052 students included in the analysis, when testing main effects, girls had lower odds for being in the Healthy Fitness Zone (HFZ) for aerobic capacity than boys. Additionally, the environment in which physical education classes take place, whether coeducational or single gender, may also affect the participation and performance of female students (38). Understanding these factors is important for informing PA programming and promoting gender-inclusive approaches to physical education.

Conversely, no significant gender differences were observed for the psychological variables, indicating that gender may not play a significant role in shaping ASE, MD, EI, SE, or SS among junior high school students. Nevertheless, other research findings differ from this research. For instance, the ASE of junior high school students showed gender differences, with some studies indicating that female students report significantly higher self-efficacy in elementary school compared to males but experience a substantial drop in self-efficacy in middle school, leading to significantly lower levels of reported self-efficacy for females than males. This gendered pattern of drop-off occurs consistently across racial groups (39). Zhang et al. (8) suggested that SS has been found to negatively correlate with depressive symptoms, with SE serving as a mediator. Gender moderates the relationship between online SS and SE, with the effect being more pronounced among males. Adak and Sarkar (11) indicated that males exhibit higher levels of internal locus of control and resilience, whereas females demonstrate lower SE. Alispahic and Hasanbegovic-Anic (17) also highlighted the role of gender in moderating the protective effects of MD. However, the absence of gender differences in psychological variables contrasts with some studies reporting gender disparities in EI and SE. This discrepancy may be due to the relatively egalitarian educational environment in Shanghai, which might mitigate gender-based psychological differences.

In this research, depression levels showed significant effects on all psychological variables (ASE, MD, EI, SE, and SS). Specifically, students with low depression scored significantly higher across these variables than those with high depression, with effect sizes (η^2^) ranging from 0.11 to 0.25, indicating medium to large effects. This finding highlighted the pervasive influence of depression on various aspects of psychological wellbeing. The findings of this study were in lined with the previous research revealed that motivational deficits are another defining characteristic of depression, leading to decreased engagement in everyday activities, including work, education, and social events. This inactivity often initiates a self-reinforcing cycle, where reduced participation worsens depressive symptoms, further lowering motivation (28). Previous research underscored the multifaceted impact of depression on psychological variables and its interplay with other factors. For instance, depression has been shown to negatively influence ASE and achievement, underscoring its detrimental role in motivational and academic domains (40). Similarly, adolescent depressive symptoms significantly impact the self-efficacy network, reducing the integration of self-efficacy and revealing the complex interconnection between these variables (41). These findings emphasized the necessity of addressing depression to enhance students' psychological and academic functioning.

Moreover, ASE emerges as a critical mediator in various contexts. It mediates the relationship between anxiety and learning burnout and between depression and learning burnout, as demonstrated in a study among nursing undergraduates in Jiangsu Province, China (42). The mediating role of ASE is further supported by research exploring its capacity to buffer depressive symptoms and smoking susceptibility, suggesting that enhancing self-efficacy could lead to more effective adolescent interventions (43). The role of contextual factors cannot be overstated, good quality environments—marked by adequate SS, effective management of school bullying, and mitigation of family conflicts—are essential for fostering EI and resilience in adolescents, ultimately equipping them to cope with challenges (44). Resilience and self-efficacy also partially mediate the associations between anxiety, depression, and academic burnout, highlighting their protective roles (45).

Additionally, academic variables and media use provide further insights into the dynamics of depression. Depression negatively predicts ASE while SE and satisfaction with studies act as protective factors, reducing depressive symptoms. Interestingly, ASE positively predicts virtual media use, which, along with depression, contributes to emotional exhaustion (46). Sowislo and Orth (25) suggested that depression also strongly associated with low SE. Collectively, these studies elucidate the interconnected relationships among depression, self-efficacy, and other psychological and contextual variables. They underscored the critical need for targeted interventions aimed at reducing depression and fostering resilience and self-efficacy to improve adolescents' overall wellbeing and academic performance.

However, depression levels did not significantly affect MVPA, suggesting that PA may not be directly influenced by depression within this sample. The reason may be that PA, particularly MVPA, may be driven more by external factors—such as school schedules, extracurricular requirements, or cultural expectations—than by individual mood states like depression. In many educational settings, especially within structured environments such as those found in Shanghai, adolescents are often required to participate in regular physical education classes and organized sports regardless of their emotional wellbeing. This external structure might buffer or obscure any direct impact of depression on MVPA levels.

### 4.1 Implication

The findings underscored the critical need to address depression in school-based mental health interventions. Programs aimed at enhancing ASE, MD, EI, SE, and SS could significantly benefit students with high levels of depression. Furthermore, the gender disparity in PA suggested the importance of tailored physical education programs to encourage greater participation among girls. The lack of interaction effects suggests that interventions addressing depression and promoting psychological wellbeing can be designed without gender-specific modifications, as the patterns of influence appear consistent across boys and girls.

### 4.2 Limitations and directions for further research

While the study provided valuable insights, it was not without limitations. First, the cross-sectional design limited the ability to infer causality. Longitudinal studies were needed to explore the dynamic relationships between depression, gender, PA, and psychological variables over time. Second, the self-reported measures may introduce response bias. Future research could incorporate objective measures of PA and validated clinical assessments of depression. Additionally, cultural factors unique to Shanghai may limit the generalizability of these findings. Replicating the study in different cultural and geographical contexts would enhance the robustness of the conclusions.

## 5 Conclusion

This study examined the role of gender and depression in predicting PA and psychological outcomes among junior high school students in Shanghai. The findings revealed significant gender differences in PA, with boys engaging in higher levels of MVPA compared to girls. However, no significant gender differences were observed in psychological variables, including ASE, MD, EI, SE, and SS. This suggests that while gender may influence PA engagement, it does not significantly affect psychological factors within this population.

Depression, on the other hand, emerged as a key factor influencing psychological outcomes. Students with lower levels of depression demonstrated significantly higher ASE, MD, EI, SE, and SS, with medium to large effect sizes. These findings highlighted the pervasive impact of depression on adolescents' psychological wellbeing. However, depression levels did not significantly affect PA, suggesting that PA may operate independently of depression within this sample.

These results emphasized the importance of addressing depression to enhance psychological wellbeing among adolescents. Interventions aimed at fostering ASE, MD, EI, SE, and SS could be particularly effective in mitigating the adverse effects of depression. Furthermore, promoting gender-inclusive approaches to PA programs may help narrow the gender gap in PA levels. Future research should explore the underlying mechanisms linking psychological variables, PA, and depression, as well as the potential for culturally tailored interventions in diverse educational contexts.

## Data Availability

The original contributions presented in the study are included in the article/Supplementary material, further inquiries can be directed to the corresponding author.
